# Who Cares for Children?

**Published:** 1977

**Authors:** S. D. M. Court


### Who Cares for Children?

S. D. M. Court*

A Foundation Lecture has a special purpose. It provides a pause in which we can pay tribute to an institution and, in doing so, consider present responsibilities in terms of past experience.

Although titled 'Who Cares for Children', it is really about those who care for the health of children.

Man is man because he asks questions; the question in this lecture has been with us from our beginning as a human species, and will remain with us if we can acquire the wisdom to survive.

My search for answers began in Birmingham in 1932 with the scientific humanity of Leonard Parsons; it found its main direction in James Spence's Newcastle where, for fifteen of my twenty-seven years there I crossed almost daily the frontier between home and hospital to make some 3,000 visits to every kind of family in every circumstance of life. Then clinical and academic colleagues brought the family and society into medical education; and the last three years have been spent moving from a room at the Department of Health with members of a many-sided committee (Department of Health and Social Security, 1976) to visit people and institutions all over the country who care for children. In this lecture, I shall take not a collective or committee but a personal and reflective view of this experience.

In this the central question becomes a quartet.

*Who are we caring for?*
*Who are the care-takers?*
*How well are they caring?*
*How could their caring be improved?*

I want you to keep these questions in mind because I don't propose a neat succession of answers; life is too untidy for that.

### WHO ARE WE CARING FOR?

We are caring for nearly 13 million children and adolescents, a quarter of our fellow citizens. But who are children and what is a child? The answer is a succession of images: an unknown unborn, a welcome newborn, a nonconformist toddler, a young schoolboy scrubbed and polished for a family visit, older school children at work and play, the vital sometimes violent, contradictory yet attractive, adolescent.

*Originally given as the Second Southmead Foundation Lecture, 5th April 1977.

I make no apology for starting with the familiar because familiarity has been our undoing. For too long the word children has concealed the fact that each stage of childhood has specific needs and calls for specific services to meet them. Man is the only species who has chosen prolonged immaturity and open-mindedness as his strategies for development. Development is the essence of childhood, and our failure to work within a developmental context has been the main cause of our restricted understanding and fragmented services.

### WHO ARE THE CARETAKERS?

### PARENTS AND FAMILY

The effective rearing of the young is a fundamental issue for all human societies. The evidence still suggests that there is no better way to raise a child than to reinforce the ability of his parents or parent, whether natural or substitute, to do so.

The family remains the primary guardian of children's health. Yet like *children* the word *family* is a difficult one, and we cannot use it as if it had a single, self-evident and accepted meaning. Let us examine it with the artist's insight. The central feature of many mediaeval paintings is the ideal mother with father, if present at all, a shadowy figure in the background.

With the development of secular society the mother and child become a family with father the dominant figure; the children, dressed as little adults, are the symbol of his social standing and success. Today, sculpture has proved more perceptive than painting and one artist, Henry Moore, in particular has been quick to recognise the growing equality and partnership of contemporary parenthood and family life, in for example, his 'Family Studies'.

What then are the characteristics of the contemporary family in which children grow and health services are used?

Marriage is more popular: more than 90 per cent of women have married by the time they reach the end of the child-bearing period. It is taking place earlier; in the last 25 years the average age for men has fallen from 27 to 24 and for women from 24 to 22.

There has been a sharp fall in the birth rate from

93 per 1000 women aged 15—44 in 1964 to 72 per 1000 in 1974. Younger marriages have not brought an increase in family size, and present trends suggest stabilisation at a completed mean family size of 2. We can therefore expect stability of the child population at present levels by 1981. And a million fewer children than 5 years ago could make possible an improvement in services without increasing cost.

Four aspects of family life today deserve special notice:

1. SINGLE PARENT FAMILIES
   Not all children live in 2 parent families; in Britain in 1971 1 million children were living in 620,000 one parent families, of which 520,000 were fatherless and 160,000 motherless (in only 10% of the fatherless families was the mother unmarried).
2. WORKING MOTHERS
   40% of mothers aged between 25 and 34 are working outside the home and this will have risen to 66% by the time the children leave school. There is still a tendency to regard the employment of mothers of young children as something that will decrease, when the indications are that we share with all other industrial countries an increasing trend for women to begin work when their children are under school age.
3. PARENTAL ILL HEALTH
   We found in our Newcastle studies (Miller et al, 1974) that 15 per cent of our mothers had psychiatric disorders or excessive degrees of weariness and depresssion. Twenty years later in an inner London Borough, Brown (1975) found a similar figure, and has shown that this malaise of motherhood varies with age and social circumstances: 25% of working class mothers compared with 5% of middle class mothers had these symptoms, and the figure rose to 42% in working class mothers with a child under five.
4. DIVORCE
   If marriage is more popular divorce is more popular too. There has been a slow upward trend in the divorce rate since 1857 when civil divorce first became available, although up to 1940 the number each year never exceeded 10,000. Since the second world war the rise has been more rapid, 58,000 in 1970 and 120,000 in 1975. And there is a parallel tendency for husbands and wives to divorce at younger ages and after shorter periods of marriage. Marriages where the bride is under 20 are twice as likely to end in divorce than when she is between 20 and 24 years. Divorce is a critical but often late event in a process of family breakdown. The needs of the separating parents are loudly proclaimed; little attention has been paid to the long-term effects upon the children, or to the effect on them of living in households where there is excessive quarrelling or violence. This situation has major implications for the health of children, and emphasises the need to admit our ignorance and to provide resources for sustained research which could discover ways of improving the quality of parenting and of strengthening counselling services to families before breakdown is imminent.

   This points more strongly than ever to the necessity for family as well as personal diagnosis, and it is disappointing that the simple but dependable system of family assessment which emerged from the Newcastle Family Studies has not been more widely used (Miller et al, 1960). It becomes of immediate importance if we aim at increasing the care of sick children in the home.

YOUNG FAMILIES

With the decline of the extended family many young mothers discover they cannot cope alone and seek local help. At present this is provided by a bewildering variety of sources: young mothers groups, pre-school play groups, day nurseries, registered (or more often unregistered) child-minders, nursery classes, nursery schools, health visitors and child health clinics.

Mutual help is to be encouraged and variety can be an advantage. However in the urban communities where most children live local authority provision is often deficient or lacking, leaving many working mothers with no alternative but child-minders of uncertain quality. Expansion and coordination of the day-care services for young children is an urgent need in our society, and its solution calls for the sustained collaboration of parents, teachers, child-minders, health visitors and social workers in each district.

ADOLESCENTS

In one sense adolescents are the healthiest, in another the most disturbed of our citizens. The range of their need is wide: accidents, cancer, sexual confusion, pregnancy (the children who are having children: 1,600 births and 3,000 abortions in girls between 11 and 16 in 1974), sexually transmitted disease (600 new cases of gonorrhoea in adolescents under 16 and nearly 4,000 between 16 and 17 in the same period), drugs especially tobacco and alcohol, and psychiatric disorders.

Many need help, yet caught in the turbulence of the change from child to adult they are often unwilling to accept it, especially from conventional authority. There is no single answer, only a series of helping hands which may or may not be taken.

There should certainly be straightforward welcoming opportunities for self-referal to school counsellor, school nurse and school doctor, but each must be trained for the work and willing to operate outside their usual setting. Where this is rejected, and at present this is so for the majority, the easy access and privacy they seek can best be met by Walk-In Advice Centres. Members of the Child Health Services Committee were familiar with a variety of these and included some in its enquiries. One of these was particularly impressive in its combination of unadvertised availability and unobtrusive professionalism.

Situated above a shop in an area of a city where young people congregate, it provided easy access to a friendly receptionist, and an unlabelled professional team consisting of a health visitor, two experienced but still youthful clinical medical officers and a child and adolescent psychiatrist. Medical examinations and advice as well as personal counselling was therefore available; 1,800 girls have been seen in the past ten years and increasingly the boy friends are coming too.

The Committee's response to such an experiment was expressed in the following statement: 'We believe there are strong grounds for thinking that if knowledge and advice were readily available in an acceptable form much distress and illness in adolescents could be prevented. We therefore commend both further epidemiological study of the pathology of adolescence and the scientific evaluation of a demonstration pilot walk-in counselling service (on the lines of the students' health service) before resources are invested in the wider development of such services'. This is an area of major need in medicine, education and social work and both courage and caution are needed in dealing with it.

### PARENTS, NEWBORNS AND PERINATAL CARE

I turn now from the end of childhood to the beginning — to the foundations of child health and family relationships.

One of the most significant advances in the care of children in my professional lifetime has come through the partnership of obstetricians, paediatricians, midwives and nurses providing special newborn care. A pregnant woman expects to be conducted safely and courteously through pregnancy and to give birth to a live and healthy baby.

For the majority this expectation will be fulfilled, but for a minority, equally precious, this will not be so. For the present, Medicine and Government have accepted the principle that all babies should be born in hospital; and I would add, on condition that the hospital can provide necessary skills in ressuscitation and newborn care. No delivery is normal until it is over. What then should be our priorities? First to meet the basic maternal need the setting should be as homely as possible: In the British Births Survey (Chamberlain et al 1970) only 37% of babies were nursed beside their mother, and the majority were in ward nurseries. There must be 24-hour provision of trained paediatric and nursing care in the special care baby units of all district general hospitals — and this will be necessary for 1 in 6 newborn babies. And for this to be possible with present or realistic future staffing we must continue to ask obstetric colleagues to arrange for mothers with greater risks of perinatal problems to be delivered where these facilities are available. Some 2—3% of babies, often the most precious of all, will require more experienced and more intensive care. This should be provided in all Regional Centres, and, progressively, in the larger district hospitals in each Area. The reasons were convincingly given in 1971 (Department of Health and Social Security); but from a combination of professional ignorance of the advances in scientific understanding, persistent but unjustified public anxiety and the administrative habit of willing the end but not providing the means, their implementation has been slow and uneven.

It is not the new techniques which should provide the necessary stimulus but the new understanding of foetal and newborn physiology which they serve. This has led to a complete reversal in the outlook for smaller low-birth weight babies. Twenty-five years ago Drillien, in an impressive study, revealed that the majority of low birth–weight babies who survived suffered substantial physical or mental impairment. This finding still dominates the conscious or unconscious thinking of many people.

But with the quality of newborn intensive care provided in this hospital and in a number of comparable centres, 9 out of 10 such babies will be within the normal range of physical and mental development. With a falling birth rate the provision of such care becomes more important than ever.

Intensive care need not prevent, as it did in the past, the early contact between mother and baby on which later maternal confidence depends. And with the timid touch in the incubator will go the photograph in the handbag, the regular news of progress, the early visits and the welcoming sister.

There is another reason why I have stressed the significance of the beginning of life. Happy expectant mothers in an antenatal clinic do not know that 1 in every 50 babies is born with a substantial malformation. Here too prenatal diagnosis, although in its early days, is beginning to make prevention possible. And for the majority there will be the joy of nourishing and protecting a growing person and a new relationship.

In the 'Doctor's Dilemma' Shaw noted that every important advance in medicine was rediscovered every 25 years. We have always known the nutritional superiority of breast milk; we are rediscovering the importance of breast feeding for bonding between mother and child; we now know that it is the best means we have of preventing early infant death and severe respiratory infection. In the antenatal and early postnatal periods therefore professional indifference must be replaced by professional understanding leading to parental explanation and support. For the second time in my professional lifetime breast-feeding matters.

**HEALTH FOR EDUCATION**

Birth is an early milestone on the developmental journey which we call childhood. Safely over, a new and accelerating phase begins — infancy, 'under-five', and then the school years to sixteen or beyond. What has paediatrics to contribute to education? The intermittent visit of school doctors to exclude physical disability or disease and school nurses seen as sources of first aid is not enough for the needs of today.

Educational failure to thrive is as significant as physical failure to thrive; it calls equally for precise diagnosis in which medical as well as educational, psychological and social understanding and skills are involved. There has never been greater need for a school health service using doctors and nurses, trained for today's problems, and working with teachers, psychologists and parents in the school and fully accepted as members of the staff.

While there are many in the present service who fulfill this with impressive skill too often they do so in isolation.

I stressed at the beginning the changing bands in the developmental spectrum of childhood. The spectrum is also a continuum, and it was this which convinced the committee that the basic paediatric component of school medicine should be provided logically by the primary care team — by the general practitioner paediatrician acting as the continuing school doctor to particular schools and working in close partnership with a well-trained school nurse.

Advice and support would come, as at present, from experienced clinical medical officers (in the Committee's terminology *clinical specialists in paediatrics*) and progressively in future from them and from consultant paediatricians trained in developmental and educational paediatrics.

### WHO CARES WHEN CHILDREN ARE ILL?

**PRIMARY CARE**

The answer to this question depends on the kinds of illness with which children and families are faced and for which they need help. These are changing; and doctors and nurses are finding it difficult to grasp what is happening and to adapt their attitudes and practice to cope with the new situation. There is still a great deal of acute illness and injury but as the tide of infective illness recedes the rocks of malformation, physical and mental handicap, chronic illness, psychiatric disorders and the 'dis-ease' in children which reflects the tensions and breakdown in family life are more sharply defined. And who would claim to be equal to the demands they make on children, parents and professionals alike?

PARENTS

The first in the chain of care are the parents and they should be encouraged to accept primary responsibility for their children's health. At the same time they cannot go it alone and need to see themselves and to be professionally accepted as partners in a 'primary care team'.

Within this a key person, perhaps the key person, is the *Health Visitor.* In 1974 there were 9,137 HVs in England and Wales, or 6,901 if we exclude those working as school nurses. This is still 50% below the level recommended by the Jameson Committee 20 years ago.

The strain of an excessive work load is intensified by underlying uncertainty about their professional role and about priorities within it. Their attachment to General Practice has made the Primary Care Team possible, but it has taken them increasingly away from children: In 1963 they were seeing 93 per cent of children under 5, in 1974 this had fallen to 77%; and today barely 60% of health visitor's time is spent with young children and their families. One way out of this dilemma would be the development of some health visitors as *child health visitors,* giving all or the major part of their time to children and their families and combining developmental oversight, preventive education and practical guidance of mothers in nursing their children at home. Others, and with additional training this could include the present district nurses, would give comparable comprehensive care to adults and the elderly.

I have described the health visitor first in the primary care team because her professional approach, especially if a measure of territorial responsibility is restored, enables her to identify families who for whatever reason are outside the service and to bring them in, and also to take services to children as well as provide them when asked.

GENERAL PRACTITIONERS

The other key member of the primary care team is the *general practitioner.* His dilemma is the accelerating increase of medical knowledge with an increasing difficulty in maintaining an acceptable standard of care for every patient of every age. Over a considerable period of time better initial and continuing paediatric training of all general practitioners would raise their standard of paediatric care. It would not equip them for the preventive and educational, as well as therapeutic, care which the changing pattern of childhood illness requires. This the committee believed could only be achieved within general practice by the development of some general practitioners as *general practitioner paediatricians.* They would remain general practitioners and full members of the practice group, but through a special interest and extended training in child health and paediatrics they would provide a source of internal consultation and ensure, themselves, or with their partners, that developmental surveillance and prevention was provided for all children in the practice together with a modern paediatric service in nearby primary and secondary schools. Where practices are single-handed or small the preventive and educational needs could be met by the attachment of clinical medical officers as *'child health practitioners'.* The urgent critical question is: will general practice understand and respond?

The third essential member of the primary care team is a part-time but consistent social worker with a commitment to child care.

**SUPPORTING CARE**

A varying but substantial number of children will be referred or come to hospitals and some will be admitted.

In view of the nature of this lecture I shall consider the people who form the chain of hospital care.

SECRETARIES

I have been impressed in certain hospitals by the trained involvement of secretaries as the open line of first contact and on-going information. This needs careful initial preparation and a genuine involvement in the on-going education of the paediatric department. With experience they develop a remarkable understanding of the nature of childhood illness and the needs of mothers. As one said to me recently 'for parents the interval between the laboratory test and getting the result is the longest time in the world'.

THE OUT-PATIENT SERVICES

The central activity of a modern paediatric department is a combined out-patient clinic and child development centre with nurses, therapists, play-worker, voluntary helpers and paediatricians providing a setting in which children and mothers feel at home and developmental, clinical and social assessment are possible.

One of the most encouraging advances in paediatrics has been the development of regional and district 'handicap teams'. Yet although the problems may be assessed in the child development centre they must be resolved in the home. Only those intimately involved in this area of human need know the loneliness, the guilt, the despair of the parents of a handicapped child. How can they be helped to find the hopeful, the life-affirming, approach on which the child's development and their stability and fulfillment will depend?

First by being treated as the senior partners in the child's care with active involvement in the remedial process: And second by unobtrusive, and consistent professional support in the home.

Yet acute and life-threatening illness and injury persist and can be treated more effectively than ever before. The strengthening of primary care previously suggested will not diminish the contribution of the hospital but rationalise it by making referal more selective.

In the Livingston Health Centre (Stark et al, 1975) this has resulted in a fall in referals from 10 per cent to 2 per cent. This means not only more out-patients time for those who need it but could allow out-patient consultation in group practices and health centres.

With only 393 consultant paediatricians in

England and Wales for 13 million children how can this be done? The services of hospitals should become more flexible, with day care, 5 day wards, and in inner city areas a realistic use of accident and emergency departments.

Yet in 1975 a million children were admitted: Who looked after them?

PARENTS

For the young child the mother, father or a close relative is needed in the hospital — resident or freely visiting. It is more than fifty years since James Spence brought mothers into hospital to share in the care of their child. In 1959 its importance was reaffirmed by the Platt Committee (Ministry of Health, 1959), yet in a residual minority of hospitals professional and administrative insensitivity to the personal needs of children and parents still denies this elemental right.

NURSES

The same Committee stated as an urgent priority: 'Sick children should be nursed by nurses who have been trained to do so'. In a sample survey of the whole country in 1974 41% of the nurses in children's wards were qualified RSCN and 22% of the enrolled nurses had had some paediatric training. The total staffing position is even more disturbing. In the words of a survey of three hospital regions in 1973 'Most children's wards are without a trained children's nurse for some of the time, many are without for much of the time, and some are without all the time'. Always on the threshold of safety children's nursing services are too often dangerously below it.

ALLIED SPECIALISTS

Half the children admitted to children's wards are admitted for surgery, and the major part of this is carried out by general or specialist surgeons whose main work is with adults. And almost as many children of school age are admitted to orthopaedic as to paediatric beds.

I have chosen a paediatric surgeon as my representative of this important area of child care, as a tribute to Arthur McPherson's service to this hospital and of his sincere promotion of what he would call pure paediatric surgery, and of your recognition of his foresight in a recent appointment. At this point I am reminded of the bald man who went to the chemist and asked once again: 'Have you anything for baldness? ' 'Nothing' replied the chemist. 'Nothing at all' ' said the anxious customer, 'Nothing but respect Sir', came the considerate reply.

Time allows me nothing but respect for the paediatric specialties (the committee was convinced of the need for their clear establishment and an increase in their numbers), and for the work of colleagues in dentistry, ophthalmology, otolaryngology, radiology, dermatology, pathology, biochemistry, microbiology and genetics.

Paediatricians work so closely with child psychiatrists that I feel they are one with us and we with them.

I would simply say that they are increasingly ready to accept, that some paediatric training should be a requirement for all specialists with a substantial commitment to children.

THERAPISTS

Doctors and nurses however competent can't go it alone. The team has come to stay and comprehensive health care means that it will grow.

Physiotherapists, speech therapists, occupational therapists, orthoptists, play-teachers, and dental auxiliaries are members of independent professions who play an increasingly important part in the care of children. And for them too the basic formula for child care holds good — child centred, parent involved, and with the professional providing not only treatment but education and support.

### THE FINAL RESPONSIBILITY

Where does the ultimate responsibility for health services for children lie? — with the public and with government — that is with us. 'Health is purchasable' and every country has the services it is able and, more important, it is willing to provide.

The main difference between Britain and our more successful neighbours Sweden, Denmark, France, is that children are higher in their scale of national priorities than our own.

The report of the Committee on Child Health Services has presented the public, the professions and government with an opportunity and a challenge: Will they take it? The next twenty-five years will show.

*Professor Court is Emeritus Professor of Child Health, University of Newcastle-upon-Tyne*

**REFERENCES** *please see page 14*

*continued from page 8* **Who Cares for Children?**

**REFERENCES**

BROWN, G. W., BHROLCHAIN, M. N. and TIRRIL, H. 1975. Social class and psychiatric disturbance among women in an urban population. *Sociology*, **9**, 225—254.

CHAMBERLAIN, R., CHAMBERLAIN, G., HEWLETT, B. and CLAIREAUX, A. 1975. *British Births 1970.* (1st Edition). **1**, London: Heinemann.

MILLER, F. J. W., COURT, S. D. M., WALTON, W. S. and KNOX, E. G. 1960. Growing up in Newcastle-upon-Tyne. (1st Edition). London: Oxford University Press.

MILLER, F. J. W., COURT, S. D. M., KNOX, E. G. and BRANDON, S. 1974. The School Years in Newcastle-upon-Tyne. London: Oxford University Press.

MINISTRY OF HEALTH. 1959. The Welfare of Children in Hospital. London: HMSO.

DEPARTMENT OF HEALTH AND SOCIAL SECURITY. 1971. Report of the Expert Group on Special Care for Babies. *In* Reports on Public Health and Medical Subjects, 127. London: HMSO.

1976. Fit for the Future. Professor S. D. M. Court. London: HMSO.

STARK, G. D., BASSETT, W. J., BAIN, D. J. G. and STEWART, F. I. 1975. Paediatrics in Livingston new town: evolution of a child health service. *British Medical Journal*, **4**, 387—390.

